# Opisthorchiasis and cholangiocarcinoma in Southeast Asia: an unresolved problem

**DOI:** 10.2147/IJGM.S133292

**Published:** 2017-08-10

**Authors:** Thomas Hughes, Thomas O’Connor, Anchalee Techasen, Nisana Namwat, Watcharin Loilome, Ross H Andrews, Narong Khuntikeo, Puangrat Yongvanit, Paiboon Sithithaworn, Simon D Taylor-Robinson

**Affiliations:** 1 Division of Digestive Health, Department of Surgery and Cancer, Imperial College London, London, UK; 2 Department of Biochemistry, Faculty of Medicine, Liver Fluke and Cholangiocarcinoma Centre; 3 Cholangiocarcinoma Screening and Care Program (CASCAP), Khon Kaen University, Khon Kaen, Thailand; 4 Faculty of Medicine, St Mary’s Campus, Imperial College, London, UK; 5 Department of Surgery; 6 Department of Biochemistry; 7 Department of Parasitology, Faculty of Medicine, Liver Fluke and Cholangiocarcinoma Centre, Khon Kaen University, Khon Kaen, Thailand

**Keywords:** *Opisthorchis viverrini*, CCA, Thailand, Laos, treatment, parasite, carcinogen, public health, helminth

## Abstract

The prevalence of cholangiocarcinoma (CCA) in Southeast Asia is much higher than other areas of the world. Eating raw, fermented, or undercooked cyprinid fish, infected with the liver fluke, *Opisthorchis viverrini* sensu lato (sl), results in chronic biliary inflammation, periductal fibrosis, and increased cancer risk. There may be associated glomerulonephritis. The process of infection is difficult to disrupt because eating practices have proven extremely difficult to change, and the life cycle of the fluke cannot be broken due to high prevalence in canine and feline reservoir hosts. Fecal analysis and enzyme-linked immunosorbent assay tests can be used to diagnose opisthorchiasis. Diagnosis of CCA is complex, partly due to the lack of definitive imaging characteristics but also due to the difficulty of obtaining samples for cytology or histology. This cancer has proven to be resistant to common chemotherapy treatments and so the two avenues of treatment available are surgical resection and liver transplantation, both requiring early detection of the tumor for the best chances of success. Late presentation of symptoms reduces the chances of successful surgical intervention. While liver fluke infections can be treated with praziquantel, individuals will often become reinfected, and multiple reinfections can be more harmful than a singular, long-term infection. A key research on the detection and characterization of novel biomarkers in all parts of the carcinogenic pathway for early diagnosis is needed.

## Biology of Opisthorchis viverrini

**Figure 1:**
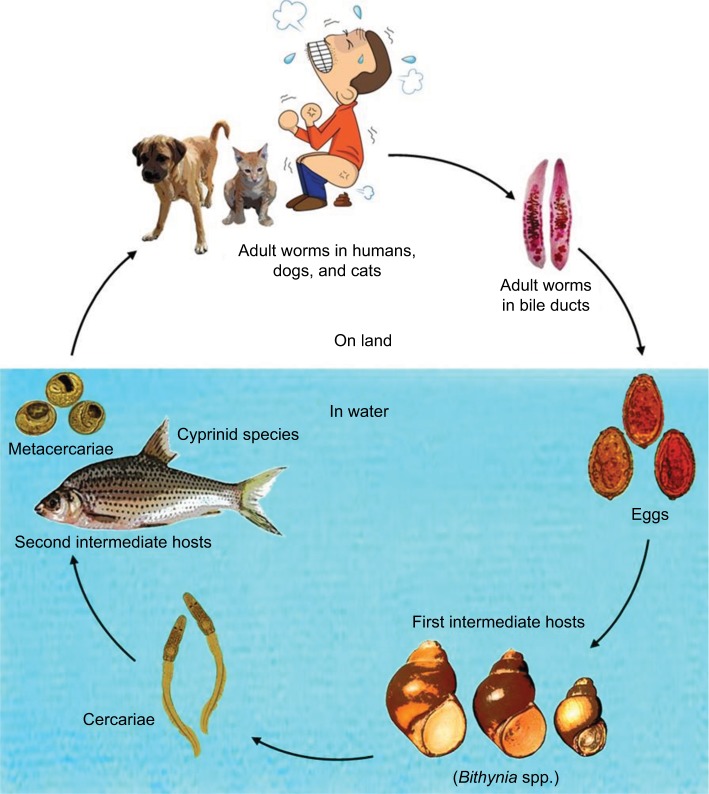
Life cycle of *Opisthorchis viverrini* sensu lato. **Note:** Courtesy of Nadda Kiatsopit.

**Figure 2:**
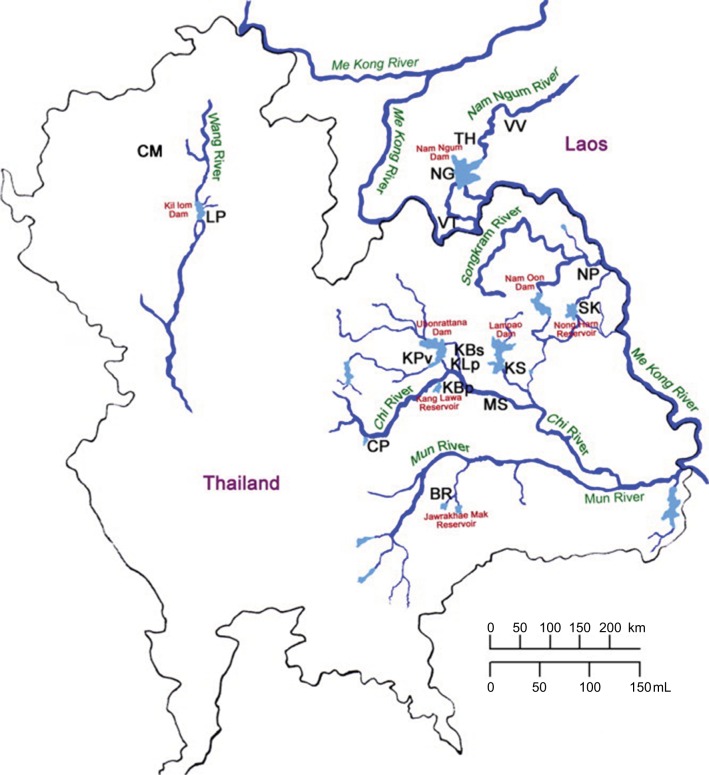
A map of river wetlands showing the geographical distribution of cryptic species of *Bithynia* snails. **Notes:** Table 1 lists the details of locations marked by abbreviations. Reproduced with permission from Saijuntha W, Sithithaworn P, Wongkham S, et al. Evidence of a species complex within the food-borne trematode Opisthorchis viverrini and possible co-evolution with their first intermediate hosts. *Int J Parasitol*. 2007;37(6):695–703.^3^

*Opisthorchis viverrini* sl is a human liver fluke that currently infects 10 million people in Northeast Thailand and in Lao Peoples’ Democratic Republic.^1^,^2^ It is a species complex, comprising many genetically distinct cryptic species (some of which are morphologically identical) that infect different genetic populations of snails, associated with specific river wetlands. Thus, it is referred to as *Opisthorchis viverrini* sensu lato (sl), or *O. viverrini* sl.^3^,^4^ The first intermediate hosts in the life cycle (Figure 1) are *Bithynia* snails, a species complex comprising at least 11 cryptic species corresponding to *O. viverrini* sl. Hence, there is a high degree of genetic variation in both species complexes associated with geographical location (such as between the Chi River wetlands and the Nam Ngum River wetlands)^5^ (Figure 2; Table 1).

These snails, *Bithynia siamensis goniomphalos*, *Bithynia siamensis*, and *Bithynia funiculata* (Figure 1), are the first intermediate hosts of *O. viverrini* sl, up to 8.37% of which can be infected, although in most regions it is <1%.^5^–^8^ Once the mature cercariae (larval trematode worms) have developed, they exit the snail host into the water and actively seek the secondary intermediate host, fish of the *Cyprinidae* family.^9^ Over 3 weeks, the cercariae develop to become infectious metacercariae (encysted larva).^10^–^12^ The fish accumulate infectious metacercariae, particularly in the head and fin regions, and have a much higher prevalence of infection, ranging from 42.9% (in *Henicorhynchus lineatus*) to 100% (in *Amblyrhynchichthys truncates*).^13^ Humans may become infected when they eat infected cyprinid fish without heating the flesh sufficiently in raw, partially cooked, or fermented dishes such as *Koi Pla*.^14^,^15^ Dishes such as this are socially and culturally significant, making the encouragement of changes to eating patterns challenging.^16^ Reinfection is therefore commonplace despite the ease of treating infection using praziquantel.^17^

Ingested metacercariae hatch out in the duodenum before moving through the common bile duct to the distal bile duct, where they mature over a period of 2 months.^10^,^11^ Canines and felines function as reservoir hosts. Cats seem to have a higher prevalence of infection than dogs (36.4% as opposed to 3.8%), and this may be to do with the method of transmission.^18^ These animals are often fed leftovers (fitting in with the traditional lifestyle of families eating raw fish), and cats appear to have a greater tendency to eat the fish heads and fins left for them.^18^ Adult liver flukes produce eggs in the gall bladder, bile duct, and pancreatic duct, which enter the digestive system in the bile and are expelled along with feces. Fecal contamination of water containing *Bithynia* snails is a big problem and causes completion of the life cycle (Figure 1). Although poor sanitation may be a major cause of human feces entering freshwater, improvements will likely not prevent the continuation of *O. viverrini* sl transmission, as the feline and canine reservoir hosts will continue to pass on the eggs (Figure 1).

## *Opisthorchis viverrini*-induced cholangiocarcinoma (CCA) pathway

The World Health Organization has classified *O. viverrini* sl as a group one carcinogen for its role in inducing CCA.^19^,^20^ Initially, liver fluke infections produce acute inflammation of the large hepatic bile ducts and portal connective tissue.^21^ However, chronic infections and inflammation have been shown to be risk factors for the development of multiple stages of carcinogenesis.^22^ Despite the ease of treatment with and mass distribution of the anti-helminthic drug, praziquantel, multiple reinfection is common and infections tend to be chronic.^15^

In chronic infections, hyperplasia, adenomatous formations, and granulomatous inflammation can be seen in the bile duct epithelium.^21^ These are caused by mechanical injury and fluke metabolic products. Oral and ventral suckers of the fluke cause mechanical damage during the processes of feeding and migration, leading to ulceration of the periductal tissue. This allows fluke eggs to enter the tissue, which in turn cause granulomatous inflammation of the tissue around them.^15^ Fluke metabolic products may be toxic or immunogenic and can interact with the bile duct epithelium to induce hyperplasia.^23^ Infiltration of inflammatory cells has been linked to the presence of *O. viverrini* sl antigens, including in areas where the liver flukes themselves are not found (eg, bile ducts too small in diameter for the flukes to enter).^15^,^21^ It has been suggested that this inflammation could be mediated by parasite-specific inflammatory cytokine interleukin (IL)-6 and later it was demonstrated that elevated IL-6 to *O. viverrini* sl excretory/secretory substance levels were linked to an increased risk of 63% for developing advanced PDF (a symptom of chronic *O. viverrini* sl infection and indicator for high risk of CCA development) and that individuals in the third quartile of IL-6 production had a 127% higher risk of developing advanced PDF than individuals in the first quartile of IL-6 production.^17^ It is now well established that chronic inflammation caused by long-term or repeated *O. viverrini* sl infections plays a large part in the development of many of the factors and events that lead to CCA, most notably the increased production of nitric oxide (NO).^24^–^27^

NO, as well as other oxygen radicals, such as superoxide (O_2_^−^), are produced in chronically inflamed tissues (using inducible NO synthase, or iNOS, in the case of NO) as an immune response aimed at killing the parasite.^25^ Parasite-specific T cells and cytokines activate cells such as macrophages, mast cells, eosinophils, and epithelial cells to synthesize NO, which is both cytotoxic (cell damaging) and genotoxic (damaging to DNA).^28^ This is largely as a result of the formation of reactive oxygen species and reactive nitrogen species (ROS/RNS). NO can react with superoxide to produce peroxynitrite (ONOO^−^), a highly reactive molecule that can cause oxidative and nitrative DNA damage through the formation of DNA adducts such as 8-oxo-7,8-dihydro-2′-deoxyguanosine (8oxodG) and 8-nitroguanine.^26^ This resultant DNA damage has been linked to an increase in cell proliferation caused by proliferating cell nuclear antigen (PCNA) accumulation in the bile duct epithelium (especially with repeat infections).^29^ As well as this, NO has been shown to further increase the possibility for carcinogenesis through DNA repair inhibition and inhibiting apoptosis.^30^–^32^ Excess production of NO, as a result of long-term *O. viverrini* sl infection and inflammation, may also increase the potential for the endogenous synthesis of N-nitrosamines, such as the carcinogenic N-dimethylnitrosamine (NDMA). This reaction has been shown to occur within the inflamed bile duct.^28^,^33^,^34^ NDMA levels have been associated with a lymphoproliferative response to *O. viverrini* sl antigens and can be detected in the urine of infected individuals.^35^

Another effect of inflammation-linked ROS/RNS is lipid peroxidation. Products of the oxidization of lipids (such as trans-4-hydroxy-2-nonenal) can react with DNA bases to form the etheno-DNA adducts 1,N^6^-etheno-2′-deoxyadenosine (εdA) and 3,N^4^-etheno-2′-deoxycytidine (εdC).^36^ These lesions can initiate carcinogenesis through specific base pair substitutions and have been shown to accumulate in the white blood cells of *O. viverrini* sl-infected individuals, alongside 8-oxodG.^36^ The presence of the DNA adducts, 8oxodG and 8-nitroguanine, in the bile duct epithelium and the presence of NDMA in the urine of *O. viverrini* sl-infected individuals cease with praziquantel treatment, strongly pushing the link between these effects and *O. viverrini* sl infection.^35^,^37^ The amount of the DNA adducts, εdA and εdC, is also significantly lowered by antiparasitic drug treatment and can also be measured in the urine.^36^ Without praziquantel treatment, 8-nitroguanine and 8-oxodG were shown to remain present in the epithelium of hamsters 180 days after they were infected.^29^ However, reinfection was shown to increase the rate of inflammatory cell infiltration, iNOS expression in the epithelium, production of NO, and formation of 8-nitroguanine and 8oxodG with each reinfection – ultimately increasing DNA damage and risk of cancer development.^29^ This raises the question as to whether or not it is best to treat individuals who are at a high risk of reinfection with praziquantel, as it could do more harm than good. There are many social and moral complications to this question, but more research into the effects of the drug and of reinfection could help come closer to finding the answer.

The diminishing immune response to a chronic *O. viverrini* sl infection may partly help to explain why multiple reinfection can be more damaging than one chronic infection, but also suggests that immunosuppression occurs.^38^ Over time, a gradual decrease in inflammation can be seen with an associated increase in PDF – one of the key stages and identifiers of risk for CCA development.^29^ Throughout the infection inflammation process, progressive destruction and remodeling of the bile duct epithelium occurs, alongside an increase in synthesis of type I and type III collagen.^17^,^39^ The linkage of PDF to increased risk for CCA development means that in Northeast Thailand, the presence of PDF is used to identify the “at-risk” group for enhanced ultrasound scanning scheduling.^40^

## Interactions between risk factors

Lifestyle-related health risks and genetic polymorphisms may interact with the effects of *O. viverrini* sl infection to enhance the potential carcinogenic effects. In animal models of *O. viverrini* sl infections, nitrosamines have been shown to be an important element of cholangiocarcinogenesis.^41^ The diet in Northeast Thailand, popularly containing fermented, dried, or salted fish and pork, is high in preformed nitrosamines and therefore may be a key cause of high CCA prevalence beyond risk of containing metacercariae.^42^ There are also frequently high levels of these in well water in Northeast Thailand.^43^ Alcohol and smoking, particularly in conjunction with one another, may enhance the carcinogenic effect of *O. viverrini* sl infection. Smoking further introduces nitrosamines into the body, alongside its many other well-known carcinogenic contents.^44^ Alcohol affects nitrosamine metabolism, distribution, and carcinogenic effect, thereby creating a greater risk of carcinogenesis for those with high nitrosamine intakes.^45^ Genetic polymorphisms such as the null variant of glutathione-S-transferase enzyme (GSTM1) may also increase cancer risk in combination with *O. viverrini* sl infection. The ineffective form of this carcinogen detoxifying enzyme is thought to lead to an increase in endogenous nitrosamine production in *O. viverrini* sl-infected individuals and an increased carcinogenic effect.^43^ There are many other biliary tract-affecting disorders that are linked to CCA incidence, such as primary sclerosing cholangitis, congenital fibropolycystic liver disease, bile duct adenomas, biliary papillomatosis, hepatolithiasis, and cirrhosis, and there are even weak links to hepatitis B and C.^46^ However, very little research published into the combined effects that these might have with *O. viverrini* sl infection exists. Another emerging risk factor for CCA is asbestos exposure.^47^ It would be interesting to explore the extent of impact this may have had in Southeast Asia, particularly in conjunction with *O. viverrini* sl infection. Further research may give greater insight into the effects that combinations of risk factors can have on the risk of an *O. viverrini* sl infection and perhaps help to shape education and policy on the matter in Southeast Asia.

## Renal comorbidity

Renal pathologies have been found to develop secondary to hepatobiliary abnormalities. In a study with hamsters infected with *O. viverrini* sl, mesangioproliferative glomerulopathy developed after 8 weeks, followed by tubular atrophy and fibrosis, as well as amyloid deposition, all coinciding with PDF and CCA.^48^ One study found immunoglobulins to *O. viverrini* sl antigens in the urine, suggestive of glomerulonephritis, and concluded IgG against *O. viverrini* sl may have potential as a biomarker due to the positive correlation with the disease progression.^49^ This is thought to be caused by the deposition of immune complexes resulting in the chemoat-traction of leukocytes and inflammation, thus increasing glomerular pore size. The greater the pore size, the more IgG reaches the urine, to such a level that it overloads the reabsorption capacity of the proximal tubule.^50^,^51^ This aspect of opisthorchiasis deserves more research to allow the disease to be correctly diagnosed and treated as a syndrome.

## Diagnosis

Formalin–ethyl acetate sedimentation concentration technique (FECT) is currently seen as the gold standard for determining *O. viverrini* sl infection and uses a microscope to count any eggs present in the feces and quantify the intensity of infection in eggs per gram (epg).^52^,^53^

FECT is a simple, noninvasive procedure, but requires an experienced microscopist, due to the frequently low egg output of individuals, and also several samples and repeats to reduce false-negatives.^52^ This method also relies on the parasites having reached the level of maturity required to release eggs into the gastrointestinal tract, which leads to a low specificity as eggs may not be present despite an *O. viverrini* sl infection. Furthermore, *O. viverrini* sl eggs can be easily confused with those of other parasites, such as minute intestinal flukes, leading to false-positives.^54^ Thus, FECT is often used alongside monoclonal antibody-based (mAb) enzyme-linked immunosorbent assay (ELISA) or indirect antibody ELISA.

mAb ELISA detects *O. viverrini* sl antigens in the feces, or coproantigens, and is more sensitive than FECT. It does not cross-react with flukes, unlike indirect antibody ELISA, but it only detects current infections, while indirect antibody ELISA can detect past infections, as antibodies persist in body fluids following treatment with praziquantel.^55^ Those with past infections are still at risk of CCA, and so indirect antibody ELISA is helpful for identifying this cohort.

Indirect antibody ELISA is used when only a blood or urine sample is available. While antibody levels are higher in serum, urine is a noninvasive alternative that does not require rapid processing or a trained phlebotomist.^56^ Both the ELISA tests are more sensitive to scanty *O. viverrini* sl infections than is FECT. The prevalence of *O. viverrini* sl infections means it is likely that some individuals have an infection with worms that have yet to reach maturity and so do not release eggs, demonstrating the need for ELISA to be used to confirm the FECT result and prevent false-negatives.^57^

The next step is to determine the extent of the disease. The presenting symptoms of CCA cannot provide a differential diagnosis, and so imaging and endoscopic techniques are often used to diagnose PDF and CCA.^58^ Ultrasound is the primary imaging tool in Southeast Asia due to its low cost and portability. It is sensitive to bile duct dilation and can detect PDF, but not malignancy. Therefore, MRI and CT scans are performed at the hospital if abnormalities are found.^59^ Both CT and MRI can determine malignancy, but MRI provides superior soft-tissue resolution, can determine periductal infiltration and the extent of localization of a tumor, and can identify small lesions to a higher degree than CT.^60^,^61^ However, CT scans cost less and so are widely used in Southeast Asia as an alternative.^62^ An endoscopic retrograde cholangiopancreatography (ERCP) is used to image the biliary tree while also obtaining biliary brushings and bile samples for histopathology and cytology.^63^,^64^ However, the fibrous nature of the tumor can lead to low yields in sample collection, and a range of other sampling and interpretive errors decrease the specificity of this technique.^65^,^66^ There is also a therapeutic value as plastic or self-expanding metal stents can be deployed to allow biliary drainage.^67^ Magnetic resonance cholangiopancreatography (MRCP) is a magnetic resonance imaging scan optimized for the biliary and pancreatic ducts and is increasingly preferred as the diagnostic test of choice due to its noninvasive nature as opposed to ERCP.^68^ Using the high water content of bile as a contrast medium, it is an excellent technique for assessing biliary obstruction and localization of lesions, but ERCP is still often needed for biopsies and deploying stents.^69^

All current available biomarkers lack specificity and sensitivity, and so the present research focuses on reliable biomarkers to allow early monitoring of opisthorchiasis.^70^,^71^

## Biomarkers

Imaging PDF is extremely subjective; hence, biomarkers are needed to determine individuals at risk of developing CCA to monitor the disease.^72^ CCA has multiple pathways, and so, ideally, multiple biomarkers are needed to cover all pathways, requiring different avenues of research.^73^

One such approach used magnetic resonance spectroscopy to analyze the chemical composition of bile in individuals with CCA against controls and assess if any differing molecules could be used as biomarkers. Using multivariate pattern-recognition analysis, CCA samples were successfully discriminated for non-CCA samples with a sensitivity of 80% and specificity of 95%, with phosphatidylcholine, H-18 bile acids, and taurine-conjugated bile acid being the most useful metabolites for differentiating the samples.^69^ Glycine-conjugated bile acids were found to be significantly raised in CCA patients’ bile, leading to the conclusion that primary bile acids, as well as glycine-conjugated bile acids, may have potential for use as biomarkers.^69^

Products caused by oxidative DNA damage can often be used as biomarkers. Hamsters infected with *O. viverrini* sl have been shown to express iNOS in the epithelial bile duct cells and inflammatory cells, resulting in an excess of ROS/RNS, and the formation of DNA adducts, such as 8-nitroguanine and 8-oxodG.^74^ The levels of the two DNA adducts were found to increase with increasing numbers of reinfections.^29^ 8-oxodG was found in leukocytes and significantly correlated with the levels in the urine and with the progression of the disease.^75^

One study found that urine 8-oxodG levels were significantly different between control individuals (healthy and advanced PDF negative – APF−) and those with advance hepatobiliary disease (CCA and advanced PDF positive – APF+). There was no significant difference between those with CCA and APF.

The area under the curve (AUC) of the receiver–operating characteristic curve to diagnose APF was 0.74 and 0.88 for CCA.^76^ Thus, 8-oxodG has been classed as robust, accurate, reproducible, and stable; however, as levels are raised in several other cancers, the ability of 8-oxodG to act as a biomarker is diminished.^77^,^78^

Immunoglobulins against *O. viverrini* sl antigens are significantly upregulated throughout the carcinogenesis process and the levels correlate with disease progression.^29^ The renal abnormalities that occur alongside the hepatobiliary pathologies mean the more advanced the disease, the higher the damage to the glomerulus, allowing more IgG to filter into the urine.^47^ One study found that >60% of individuals who were APF+, and all of the CCA individuals, tested positive for IgG against *O. viverrini* sl antigen in the urine, yet nearly all individuals had it in their blood. This implies that only individuals with advanced hepatobiliary pathologies had developed renal complications significant enough for the IgG to be present in the urine. The levels of urine IgG against *O. viverrini* sl were significantly higher in individuals with hepatobiliary abnormalities as opposed to controls, were able to differentiate between CCA and other stages of opisthorchiasis, and could discriminate between light and heavy *O. viverrini* sl infections.^48^

## DNA mutations

CpG islands are a potential source of biomarkers and are often methylated in cancers which result in transcriptional silencing.^79^,^80^ One study aimed at looking for hypermethylation at certain loci to find prognostic or diagnostic biomarkers.^81^ In order to analyze the data, a methylation index (MI) was created which was the ratio of the number of methylated CpG islands and the total number of CpG islands looked at.^82^ The result was that the MI for CCA (0.12) was significantly higher than that for normal tissues (0.02). Opioid binding protein/cell adhesion molecule like gene (OPCML) was the most commonly methylated (72.5%).^80^ It was not found methylated in normal tissues and was associated with less differentiated CCA, leading to a worse prognosis and increased chance of metastasis.^83^,^84^

OPCML is a tumor-suppressor gene and is methylated in several other cancers, such as ovarian cancer, and so is of little use when used alone.^85^ PTEN, HIC1, and SFRP1 were also all often found to be highly methylated and are thought to play a role in the increase of proliferation and survival advantage as well as having an antiapoptotic effect. Patients who had methylation at the locus of DcR1 were found to have a longer survival than those without, suggesting that DcR1 could be used to indicate the prognosis of CCA patients.^80^

## MicroRNA

Many cancers cause an up/downregulation of certain miRNAs which can be detected in bodily fluids.^86^ In CCA, the levels of several miRNAs have been found to change, such as miR-141 which increases, and miR-370 which decreases, and miR-21 which was found to correlate with the disease progression in hamsters.^71^,^87^,^88^ One study found that serum miR-192 levels in CCA patients were significantly higher than in healthy subjects, with an AUC of 0.803, but were not significantly increased as the opisthorchiasis progressed.

Despite this, the increase in miR-192 levels throughout the carcinogenesis pathway implies that the increase is caused by parasite-driven inflammation. High levels also carried a significant correlation with lymph node metastasis and decreased survival rates, meaning it can be used as a prognostic marker.^89^

The use of miR-192 as a biomarker when found in the urine has also been researched, alongside miR-21. In the urine, the miR-192 levels were significantly different between healthy and *O. viverrini* sl infected, healthy and PDF, and healthy and CCA. Not only this, but the level of miRNA-192 between healthy and other inflammatory diseases was not significant and so can be described as disease specific. miR-21 was found to be significantly higher for PDF, CCA, and other inflammatory diseases as opposed to healthy subjects, making it a useful potential biomarker, although it is not unique for CCA. When the two miRNAs were combined, the results for differentiating between individuals improved and removed the problem of miR-21 not being disease specific.^90^

## Current treatment

Praziquantel is a cheap and effective anti-helminth drug to treat *O. viverrini* sl infections and was used for mass drug administration in Thailand in the early 1980s to prevent CCA. This effectively reduced the prevalence from 80% to 15%–20% by 1997.^91^ Although praziquantel will stop parasitic infection, any inflammation and fibrosis in the bile duct that remain will be a risk factor for CCA development. A further problem is that many individuals will become reinfected with *O. viverrini* sl which may increase the risk for CCA, due to the acute inflammatory phase of reinfection, as opposed to chronic inflammation.^92^ Therefore, repeat drug administration may increase the risk of cholangiocarcinogenesis if people continue to eat raw fish and get reinfected following praziquantel treatment.

Effective CCA treatment is limited, showing a resistance to chemotherapy, with a very low response rate.^93^ One study has potentially found a future use for chemotherapy: to prevent TRAIL (Tumor necrosis factor Related Apoptosis Inducing Ligand) resistance. When used in conjunction with recombinant TRAIL, or TRAIL receptor agonistic monoclonal antibodies, it is potentially a selective CCA treatment, as tumor cells are much more sensitive than normal cells to TRAIL-induced apoptosis.^80^

Nonetheless, in Southeast Asia, the only two current effective treatments are surgical resection and liver transplantation. By the time CCA presents, the prognosis is usually poor, as the tumor is too advanced for resection. While hilar tumors can be aggressively managed with portal embolization alongside liver and portal vein resections, extrahepatic tumor growth is a contraindication for surgery. Reports show that, of those who had a “successful,” that is, R0 resection, the 5-year survival rate was still only 25%–30%, dropping to 0%–12% for patients with positive margins.^94^,^95^ A study group from Northeast Thailand managed to gain a promising R0 resection rate of 64% from 83 patients. The 5-year survival for the entire group was 21% while that of the R0 group was 30%.^96^ Despite a poor general prognosis, when neoadjuvant chemotherapy is used, a 65% cure rate can be achieved with perihilar CCA cases.^97^

When resection is not an option for intrahepatic CCA, those without any extrahepatic growth may be eligible for liver transplant.^93^ In the past, liver transplantation was seen as ineffective in treating CCA with a 5-year survival rate of 0%–20% in published series from Kings College London and Hannover.^98^,^99^ However, recent studies have given renewed interest in liver transplantation, with a US center achieving a 5-year survival rate of >70% using neoadjuvant chemotherapy with a carefully selected group.^100^ Another group in Scandinavia achieved a 50% 5-year survival rate, again with highly stringent patient selection.^101^ Typically, these cases involve very small tumors, which are deemed nonresectable, and occur on the perihilar region of the liver. Only a few percent of those considered are deemed eligible. These studies indicate that instead of seeing liver transplantation as a poor alternative, the guidelines for which patients are eligible should be more stringent.

Immunotherapy is a promising new treatment to be used adjuvantly to surgery and/or chemotherapy. The presence of leukocytes in infected sites allows for exploitation of the immune system to fight the tumor.^102^ Personal peptide vaccines, which immunize the patient against a host of antigens, and dendritic vaccines, which immunize against one antigen with no need for processing and a reduced autoimmunity are both significantly more effective used adjuvantly with chemotherapy compared to chemotherapy alone.^103^ As well as with chemotherapy, dendritic vaccines resulted in an average survival time of 17.4 months when administered alongside surgery compared to 7.7 months for surgery alone.^104^ A meta-analysis found that a higher expression of immune active factors resulted in a significantly better prognosis (*P*<0.00001) and that altering the tumor microenvironment to increase the T-cell infiltration significantly increased the 6-month progression-free survival rate (*P*<0.05).^101^ Cytotoxic T-lymphocyte antigen 4 and the interaction between programmed cell death 1 (PD1) and its ligand (PDL1) are pathways the tumor uses to evade the immune system. Passive immunotherapy uses monoclonal antibodies to block these interactions so the immune system can fight the tumor. However, while this has been done in other malignancies such as melanoma, there is no published data on the efficacy against CCA.^105^

When the tumor is too advanced for curative treatment, the only other option is palliative care. In Northeast Thailand, over 95% of cases have stage 4 CCA and so 70%–80% of patients receive palliative treatment using stents for biliary drainage.^106^,^107^ The high number of stage 4 CCA cases highlights the need for research into earlier diagnosis to find cases earlier on in their progression.

## Summary

The high rate of *O. viverrini* sl infection in Southeast Asia has led to Northeast Thailand having the highest incidence of CCA in the world. Infection with the liver fluke can be easily diagnosed with FECT or ELISA, with praziquantel being used as treatment. However, due to CCA-afflicted individuals remaining asymptomatic until the end stages of the disease, the cure rate without early diagnosis and intervention is dire. Thus, ultrasounds are now routinely used to screen individuals for potentially early stages of CCA or those in the risk group with PDF. MRI and CT scans are used further to evaluate potential malignancy and ERCPs can be used for further confirmation. *O. viverrini* sl has also been implicated in renal pathology, caused by an immune response with resultant glomerulonephritis. The resulting IgG found in urine has the potential to be used as a syndromic biomarker. Research into biomarkers from various aspects of the carcinogenic pathway is now underway to detect CCA in the early stages and allow for a higher chance of successful intervention.

## Conclusion

To best combat CCA in Southeast Asia in the short term, research should focus on early and better diagnosis of CCA and its warning signs. Continued effort to produce a range of biomarkers for various stages of the carcinogenic pathway could lead to a dramatically reduced mortality rate and better prognoses for patients. Longer-term future public education schemes, such as those organized by the Cholangiocarcinoma Screening and Care Program (CASCAP), should continue to be introduced and expanded to teach communities about the dangers of eating certain types of raw, partially cooked, or fermented fish, and perhaps over time, practices will evolve concerning “raw attitudes.”^14^ Investigation into the under-researched renal effects of opisthorchiasis could also garner some insight into the complicated effect of long-term *O. viverrini* infection and how it can be tackled as a syndrome.

## Figures and Tables

**Table 1:** Details of locations from which cryptic species of *Bithynia* have been found

Code	Collecting locality	Wetland	Province (village/district)	Country
KBs	Kang Namton Reservoir	Chi River	Khon Kaen (Ban Sa-ard)	Thailand
KLp	Prakeu Stream	Chi River	Khon Kaen (Ban Lerngpleuy)	Thailand
KBp	Kang Lawa Reservoir	Chi River	Khon Kaen (Ban Phai)	Thailand
KPv	Ubonrattana Dam	Chi River	Khon Kaen (Phuviang)	Thailand
CP	Nong Ben Reservoir	Chi River	Chaiya Phum	Thailand
MS	Chi River	Chi River	Mahasarakham	Thailand
KS	Lampao Dam	Chi River	Kalasin	Thailand
LP	Kil Lom Dam	Wang River	Lampang	Thailand
CM	Rice field near Mae Ping River	Mae Ping River	Chiang Mai (Mae Rim)	Thailand
BR	Huay Jawrakhae Mak Reservoir	Mun River	Buri Ram	Thailand
SK	Nong Harn Reservoir	Songkram River	Sakon Nakhon	Thailand
NP	Songkram River	Songkram River	Nakon Phanom	Thailand
VV	Nam Ngum Dam	Nam Ngum River	Vang Vieng	Laos PDR
NG	Nam Ngum Dam	Nam Ngum River	Nam Ngum	Laos PDR
TH	Nam Ngum Dam	Nam Ngum River	Tha Heur	Laos PDR
VT	Nam Ngum Dam	Nam Ngum River	Vientiane	Laos PDR

**Notes:** See Figure 2 for locations. Reproduced with permission from Saijuntha W, Sithithaworn P, Wongkham S, et al. Evidence of a species complex within the food-borne trematode Opisthorchis viverrini and possible co-evolution with their first intermediate hosts. *Int J Parasitol*. 2007;37(6):695–703.^3^

## References

[1] Keiser J, Utzinger J (2009). Food-borne trematodiases. Clin Microbiol Rev.

[2] Andrews RH, Sithithaworn P, Petney TN (2008). Opisthorchis viverrini: an underestimated parasite in world health. Trends Parasitol.

[3] Saijuntha W, Sithithaworn P, Wongkham S (2007). Evidence of a species complex within the food-borne trematode Opisthorchis viverrini and possible co-evolution with their first intermediate hosts. Int J Parasitol.

[4] Sithithaworn P, Andrews RH, Petney TN, Saijuntha W, Laoprom N (2012). The systematics and population genetics of Opisthorchis viverrini sensu lato: implications in parasite epidemiology and bile duct cancer. Parasitol Int.

[5] Kiatsopit N, Sithithaworn P, Saijuntha W, Petney TN, Andrews RH (2013). *Opisthorchis viverrini*: implications of the systematics of first intermediate hosts, *Bithynia* snail species in Thailand and Lao PDR. Infect Genet Evol.

[6] Brockelman WY, Upatham ES, Viyanant V, Ardsungnoen S, Chantanawat R (1986). Field studies on the transmission of the human liver fluke, *Opisthorchis viverrini*, in northeast Thailand: population changes of the snail intermediate host. Int J Parasitol.

[7] Namsanor J, Sithithaworn P, Kopolrat K (2015). Seasonal transmission of *Opisthorchis viverrini* sensu lato and a lecithodendriid trematode species in *Bithynia siamensis* goniomphalos snails in northeast Thai-land. Am J Trop Med Hyg.

[8] Kiatsopit N, Sithithaworn P, Saijuntha W (2012). Exceptionally high prevalence of infection of *Bithynia siamensis* goniomphalos with *Opisthorchis viverrini cercariae* in different wetlands in Thailand and Lao PDR. Am J Trop Med Hyg.

[9] Haas W, Granzer M, Brockelman CR (1990). *Opisthorchis viverrini*: finding and recognition of the fish host by the cercariae. Exp Parasitol.

[10] Sithithaworn P, Yongvanit P, Duenngai K, Kiatsopit N, Pairojkul C (2014). Roles of liver fluke infection as risk factor for cholangiocarcinoma. J Hepatobiliary Pancreat Sci.

[11] Sithithaworn P, Haswell-Elkins M (2003). Epidemiology of *Opisthorchis viverrini*. Acta Trop.

[12] Kopolrat K, Sithithaworn P, Kiatsopit N (2016). Comparison of infectivity, metacercarial burden and host mortality induced by *Opisthorchis viverrini* sensu lato cercariae from Lao PDR compared with Thailand in cyprinid fish, *Barbonymus gonionotus*. Trans R Soc Trop Med Hyg.

[13] Donthaisong C, Arunsan P, Suwannatrai K (2014). Experimental infection of *Opisthorchis viverrini cercariae* to the cyprinid fish, *Barbonymus gonionotus*. Acta Trop.

[14] Grundy-Warr C, Andrews RH, Sithithaworn P (2012). Raw attitudes, wetland cultures, life-cycles: socio-cultural dynamics relating to *Opisthorchis viverrini* in the Mekong Basin. Parasitol Int.

[15] Sripa B, Kaewkes S, Sithithaworn P (2007). Liver fluke induces cholangiocarcinoma. PLoS Med.

[16] Macpherson CN (2005). Human behaviour and the epidemiology of parasitic zoonoses. Int J Parasitol.

[17] Sripa B, Mairiang E, Thinkhamrop B (2009). Advanced periductal fibrosis from infection with the carcinogenic human liver fluke *Opisthorchis viverrini* correlates with elevated levels of interleukin-6. Hepatology.

[18] Enes JE, Wages AJ, Malone JB, Tesana S (2010). Prevalence of *Opisthorchis viverrini* infection in the canine and feline hosts in three villages, Khon Kaen Province, northeastern Thailand. Southeast Asian J Trop Med Public Health.

[19] Young ND, Campbell BE, Hall RS (2010). Unlocking the transcriptomes of two carcinogenic parasites, *Clonorchis sinensis* and *Opisthorchis viverrini*. PLoS Negl Trop Dis.

[20] World Health Organisation (2014). Biological agents–a review of human carcinogens. IARC Monogr Eval Carcinog Risks Hum.

[21] Bhamarapravati N, Thammavit W, Vajrasthira S (1978). Liver changes in hamsters infected with a liver fluke of man, *Opisthorchis viverrini*. Am J Trop Med Hyg.

[22] Mantovani A, Allavena P, Sica A, Balkwill F (2008). Cancer-related inflammation. Nature.

[23] Pungpak S, Chalermrut K, Harinasuta T (1994). *Opisthorchis viverrini* infection in Thailand: symptoms and signs of infection–a population-based study. Trans R Soc Trop Med Hyg.

[24] Holzinger F, Z’Graggen K, Buchler MW (1999). Mechanisms of biliary carcinogenesis: a pathogenetic multi-stage cascade towards cholangiocarcinoma. Ann Oncol.

[25] Ohshima H, Bartsch H (1994). Chronic infections and inflammatory processes as cancer risk factors: possible role of nitric oxide in carcinogenesis. Mutat Res.

[26] Inoue S, Kawanishi S (1995). Oxidative DNA damage induced by simultaneous generation of nitric oxide and superoxide. FEBS Lett.

[27] Kawanishi S, Hiraku Y (2006). Oxidative and nitrative DNA damage as biomarker for carcinogenesis with special reference to inflammation. Antioxid Redox Signal.

[28] Yongvanit P, Pinlaor S, Bartsch H (2012). Oxidative and nitrative DNA damage: key events in opisthorchiasis-induced carcinogenesis. Parasitol Int.

[29] Pinlaor S, Sripa B, Sithithaworn P, Yongvanit P (2004). Hepatobiliary changes, antibody response, and alteration of liver enzymes in hamsters re-infected with *Opisthorchis viverrini*. Exp Parasitol.

[30] Jaiswal M, LaRusso NF, Burgart LJ, Gores GJ (2000). Inflammatory cytokines induce DNA damage and inhibit DNA repair in cholangiocarcinoma cells by a nitric oxide-dependent mechanism. Cancer Res.

[31] Jaiswal M, LaRusso NF, Shapiro RA, Billiar TR, Gores GJ (2001). Nitric oxide-mediated inhibition of DNA repair potentiates oxidative DNA damage in cholangiocytes. Gastroenterology.

[32] Torok NJ, Higuchi H, Bronk S, Gores GJ (2002). Nitric oxide inhibits apoptosis downstream of cytochrome C release by nitrosylating caspase 9. Cancer Res.

[33] Srivatanakul P, Ohshima H, Khlat M (1991). *Opisthorchis viverrini* infestation and endogenous nitrosamines as risk factors for cholangiocarcinoma in Thailand. Int J Cancer.

[34] Satarug S, Haswell-Elkins MR, Tsuda M (1996). Thiocyanate-independent nitrosation in humans with carcinogenic parasite infection. Carcinogenesis.

[35] Satarug S, Haswell-Elkins MR, Sithithaworn P (1998). Relationships between the synthesis of N-nitrosodimethylamine and immune responses to chronic infection with the carcinogenic parasite, *Opisthorchis viverrini*, in men. Carcinogenesis.

[36] Dechakhamphu S, Pinlaor S, Sitthithaworn P, Nair J, Bartsch H, Yongvanit P (2010). Lipid peroxidation and etheno DNA adducts in white blood cells of liver fluke-infected patients: protection by plasma alpha-tocopherol and praziquantel. Cancer Epidemiol Biomarkers Prev.

[37] Pinlaor S, Hiraku Y, Yongvanit P (2006). iNOS-dependent DNA damage via NF-kappaB expression in hamsters infected with *Opisthorchis viverrini* and its suppression by the antihelminthic drug praziquantel. Int J Cancer.

[38] Sripa B, Kaewkes S (2000). Relationship between parasite-specific antibody responses and intensity of *Opisthorchis viverrini* infection in hamsters. Parasite Immunol.

[39] Chotigeat W, Ruenwongsa P (1986). Types of collagen in *Opisthorchis viverrini* infected hamster liver. Mol Biochem Parasitol.

[40] Mairiang E, Elkins DB, Mairiang P (1992). Relationship between intensity of Opisthorchis viverrini infection and hepatobiliary disease detected by ultrasonography. J Gastroenterol Hepatol.

[41] Thamavit W, Bhamarapravati N, Sahaphong S, Vajrasthira S, Angsubhakorn S (1978). Effects of dimethylnitrosamine on induction of cholangiocarcinoma in *Opisthorchis viverrini*-infected Syrian golden hamsters. Cancer Res.

[42] Srivatanakul P, Sukaryodhin S, Ohshima H (1991). *Opisthorchis viverrini* infestation and endogenous nitrosamines as risk factors for cholangiocarcinoma in Thailand. Int J Cancer.

[43] Honjo S, Srivatanakul P, Sriplung H (2005). Genetic and environmental determinants of risk for cholangiocarcinoma via *Opisthorchis viverrini* in a densely infested area in Nakhon Phanom, northeast Thailand. Int J Cancer.

[44] Tricker AR (1997). N-nitroso compounds and man: sources of exposure, endogenous formation and occurrence in body fluids. Eur J Cancer Prev.

[45] Swann PF (1984). Effect of ethanol on nitrosamine metabolism and distribution Implications for the role of nitrosamines in human cancer and for the influence of alcohol consumption on cancer incidence. IARC Sci Publ.

[46] Khan SA, Toledano MB, Taylor-Robinson SD (2008). Epidemiology, risk factors, and pathogenesis of cholangiocarcinoma. HPB.

[47] Brandi G, Di Girolamo S, Farioli A (2013). Asbestos: a hidden player behind the cholangiocarcinoma increase? Findings from a case–control analysis. Cancer Causes Control.

[48] Boonpucknavig S, Boonpucknavig V, Tanvanich S, Doungchawee G, Thamavit W (1992). Development of immune-complex glomerulonephritis and amyloidosis in Syrian golden hamsters infected with *Opisthorchis viverrini*. J Med Assoc Thai.

[49] Saichua P, Sithithaworn P, Jariwala AR (2013). Microproteinuria during *Opisthorchis viverrini* infection: a biomarker for advanced renal and hepatobiliary pathologies from chronic opisthorchiasis. PLoS Negl Trop Dis.

[50] D’Amico G, Bazzi C (2003). Pathophysiology of proteinuria. Kidney Int.

[51] Levinsky RJ (1981). Role of circulating immune complexes in renal diseases. J Clin Pathol.

[52] CDC-DPDx (2016). DPDx – Laboratory Identification of Parasitic Diseases of Public Health Concern. DPDx – Laboratory Identification of Parasitic Diseases of Public Health Concern.

[53] Johansen MV, Lier T, Sithithaworn P (2015). Towards improved diagnosis of neglected zoonotic trematodes using a One Health approach. Acta Trop.

[54] Utzinger J, Brattig NW, Leonardo L, Zhou XN, Bergquist R (2015). Progress in research, control and elimination of helminth infections in Asia. Acta Trop.

[55] Jamornthanyawat N (2002). The diagnosis of human opisthorchiasis. Southeast Asian J Trop Med Public Health.

[56] Sawangsoda P, Sithithaworn J, Tesana S (2012). Diagnostic values of parasite-specific antibody detections in saliva and urine in comparison with serum in opisthorchiasis. Parasitol Int.

[57] Worasith C, Kamamia C, Yakovleva A (2015). Advances in the diagnosis of human opisthorchiasis: development of *Opisthorchis viverrini* antigen detection in urine. PLoS Negl Trop Dis.

[58] Nakeeb A, Pitt HA, Sohn TA (1996). Cholangiocarcinoma. A spectrum of intrahepatic, perihilar, and distal tumors. Ann Surg.

[59] Zabron A, Edwards RJ, Khan SA (2013). The challenge of cholangiocarcinoma: dissecting the molecular mechanisms of an insidious cancer. Dis Model Mech.

[60] Chamadol N, Pairojkul C, Khuntikeo N (2014). Histological confirmation of periductal fibrosis from ultrasound diagnosis in cholangiocarcinoma patients. J Hepatobiliary Pancreat Sci.

[61] Wadsworth CA, Lim A, Taylor-Robinson SD, Khan SA (2013). The risk factors and diagnosis of cholangiocarcinoma. Hepatol Int.

[62] Ibrahim RSS, Mazli M, Amrizal M, Aljunid S (2012). Cost of magnetic resonance imaging (MRI) and computed tomography (CT) scan in UKMMC. BMC Health Serv Res.

[63] Khan SA, Davidson BR, Goldin R (2002). Guidelines for the diagnosis and treatment of cholangiocarcinoma: consensus document. Gut.

[64] Boberg KM, Jebsen P, Clausen OP, Foss A, Aabakken L, Schrumpf E (2006). Diagnostic benefit of biliary brush cytology in cholangiocarcinoma in primary sclerosing cholangitis. J Hepatol.

[65] Weber A, von Weyhern C, Fend F (2008). Endoscopic transpapillary brush cytology and forceps biopsy in patients with hilar cholangiocarcinoma. World J Gastroenterol.

[66] The Free Dictionary (2016). Biliary brushing.

[67] Soderlund C, Linder S (2006). Covered metal versus plastic stents for malignant common bile duct stenosis: a prospective, randomized, controlled trial. Gastrointest Endosc.

[68] Prasad SR, Sahani D, Saini S (2001). Clinical applications of magnetic resonance cholangiopancreatography. J Clin Gastroenterol.

[69] Romagnuolo J, Bardou M, Rahme E, Joseph L, Reinhold C, Barkun AN (2003). Magnetic resonance cholangiopancreatography: a meta-analysis of test performance in suspected biliary disease. Ann Intern Med.

[70] Sharif AW, Williams HR, Lampejo T (2010). Metabolic profiling of bile in cholangiocarcinoma using in vitro magnetic resonance spectroscopy. HPB (Oxford).

[71] Alvaro D (2009). Serum and bile biomarkers for cholangiocarcinoma. Curr Opin Gastroenterol.

[72] Yongvanit P, Pinlaor S, Loilome W (2014). Risk biomarkers for assessment and chemoprevention of liver fluke-associated cholangiocarcinoma. J Hepatobiliary Pancreat Sci.

[73] Kelloff GJ, Boone CW, Crowell JA (1996). Risk biomarkers and current strategies for cancer chemoprevention. J Cell Biochem Suppl.

[74] Pinlaor S, Yongvanit P, Hiraku Y (2003). 8-nitroguanine formation in the liver of hamsters infected with *Opisthorchis viverrini*. Biochem Biophys Res Commun.

[75] Thanan R, Murata M, Pinlaor S (2008). Urinary 8-oxo-7,8-dihydro-2′-deoxyguanosine in patients with parasite infection and effect of antiparasitic drug in relation to cholangiocarcinogenesis. Cancer Epidemiol Biomarkers Prev.

[76] Saichua P, Yakovleva A, Kamamia C (2015). Levels of 8-oxodG predict hepatobiliary pathology in *Opisthorchis viverrini* endemic settings in Thailand. PLoS Negl Trop Dis.

[77] Evans MD, Olin-ski R, Loft S, Cooke MS, European Standards Committee on Urinary Lesion A (2010). Toward consensus in the analysis of urinary 8-oxo-7,8-dihydro-2′-deoxyguanosine as a noninvasive biomarker of oxidative stress. FASEB J.

[78] Gabrielli A, Svegliati S, Moroncini G, Amico D (2012). New insights into the role of oxidative stress in scleroderma fibrosis. Open Rheumatol J.

[79] Illingworth RS, Bird AP (2009). CpG islands–‘a rough guide’. FEBS Lett.

[80] Esteller M (2006). Epigenetics provides a new generation of oncogenes and tumour-suppressor genes. Br J Cancer.

[81] Sriraksa R, Zeller C, El-Bahrawy MA (2011). CpG-island methylation study of liver fluke-related cholangiocarcinoma. Br J Cancer.

[82] Gutierrez MI, Siraj AK, Khaled H, Koon N, El-Rifai W, Bhatia K (2004). CpG island methylation in Schistosoma- and non-Schistosoma-associated bladder cancer. Mod Pathol.

[83] Nakajima T, Kondo Y, Miyazaki M, Okui K (1988). A histopathologic study of 102 cases of intrahepatic cholangiocarcinoma: histologic classification and modes of spreading. Hum Pathol.

[84] World Health Organisation (2000). Pathology and Genetics of Tumours of the Digestive System.

[85] Teodoridis JM, Hall J, Marsh S (2005). CpG island methylation of DNA damage response genes in advanced ovarian cancer. Cancer Res.

[86] Kosaka N, Iguchi H, Ochiya T (2010). Circulating microRNA in body fluid: a new potential biomarker for cancer diagnosis and prognosis. Cancer Sci.

[87] Meng F, Henson R, Lang M (2006). Involvement of human micro-RNA in growth and response to chemotherapy in human cholangiocarcinoma cell lines. Gastroenterology.

[88] Meng F, Wehbe-Janek H, Henson R, Smith H, Patel T (2008). Epigenetic regulation of microRNA-370 by interleukin-6 in malignant human cholangiocytes. Oncogene.

[89] Silakit R, Loilome W, Yongvanit P (2014). Circulating miR-192 in liver fluke-associated cholangiocarcinoma patients: a prospective prognostic indicator. J Hepatobiliary Pancreat Sci.

[90] Silakit R, Loilome W, Yongvanit P (2015). Urinary microRNA-192 and microRNA-21 as potential indicators for liver fluke-associated cholangiocarcinoma risk group. Parasitol Int.

[91] Jongsuksuntigul P, Imsomboon T (2003). Opisthorchiasis control in Thailand. Acta Trop.

[92] Kamsa-Ard S, Luvira V, Pugkhem A (2015). Association between praziquantel treatment and cholangiocarcinoma: a hospital-based matched case-control study. BMC Cancer.

[93] Scheithauer W (2002). Review of gemcitabine in biliary tract carcinoma. Semin Oncol.

[94] Friman S (2011). Cholangiocarcinoma – current treatment options. Scand J Surg.

[95] Jang JY, Kim SW, Park DJ (2005). Actual long-term outcome of extrahepatic bile duct cancer after surgical resection. Ann Surg.

[96] Lang H, Sotiropoulos GC, Sgourakis G (2009). Operations for intrahepatic cholangiocarcinoma: single-institution experience of 158 patients. J Am Coll Surg.

[97] Luo X, Yuan L, Wang Y, Ge R, Sun Y, Wei G (2014). Survival outcomes and prognostic factors of surgical therapy for all potentially resectable intrahepatic cholangiocarcinoma: a large single-center cohort study. J Gastrointest Surg.

[98] Ringe B, Wittekind C, Bechstein WO, Bunzendahl H, Pichlmayr R (1989). The role of liver transplantation in hepatobiliary malignancy. A retrospective analysis of 95 patients with particular regard to tumor stage and recurrence. Ann Surg.

[99] O’Grady JP RJ, Rolles K, Williams R (1988). Liver transplantation for malignant disease. Ann Surg.

[100] Rosen CB, Heimbach JK, Gores GJ (2010). Liver transplantation for cholangiocarcinoma. Transpl Int.

[101] Friman S, Foss A, Isoniemi H (2011). Liver transplantation for cholangiocarcinoma – selection is essential for acceptable results. Scand J Gastroenterol.

[102] Wang Y, Ding M, Zhnag Q (2017). Activation or suppression of the immune response mediators in biliary tract cancer (BTC) patients: a systematic review and meta-analysis. J Cancer.

[103] Marks EI, Yee NS (2015). Immunotherapeutic approaches in biliary tract carcinoma: Current status and emerging strategies. World J Gastrointest Oncol.

[104] Takahashi R, Yoshitomi M, Yutani S (2013). Current status of immunotherapy for the treatment of biliary tract cancer. Hum Vaccin Immunother.

[105] Gubin MM, Zhang X, Schuster H, Caron E (2014). Checkpoints blockade cancer immunotherapy targets tumour-specific mutant antigens. Nature.

[106] Wiangnon S, Kamsa-ard S, Suwanrungruang K (2012). Trends in incidence of hepatocellular carcinoma, 1990–2009, Khon Kaen, Thailand. Asian Pac J Cancer Prev.

[107] Martin RC, Vitale GC, Reed DN, Larson GM, Edwards MJ, McMasters KM (2002). Cost comparison of endoscopic stenting vs surgical treatment for unresectable cholangiocarcinoma. Surg Endosc.

